# To Explore the Mechanism and Equivalent Molecular Group of Fuxin Mixture in Treating Heart Failure Based on Network Pharmacology

**DOI:** 10.1155/2020/8852877

**Published:** 2020-11-21

**Authors:** Yi-ding Yu, Yi-ping Xiu, Yang-fan Li, Yi-tao Xue

**Affiliations:** ^1^Shandong University of Traditional Chinese Medicine, Jinan 250014, China; ^2^Affiliated Hospital of Shandong University of Traditional Chinese Medicine, Jinan 250014, China

## Abstract

Fuxin mixture (FXHJ) is a prescription for the treatment of heart failure. It has been shown to be effective in clinical trials, but its active ingredients and mechanism of action are not completely clear, which limits its clinical application and international promotion. In this study, we used network pharmacology to find, conclude, and summarize the mechanism of FXHJ in the treatment of heart failure. From FXHJ, we found 39 active ingredients and 47 action targets. Next, we constructed the action network and was conducted enrichment analysis. The results showed that FXHJ mainly treated heart failure by regulating the MAPK signaling pathway, PI3KAkt signaling pathway, cAMP signaling pathway, TNF signaling pathway, toll-like receptor signaling pathway, VEGF signaling pathway, NF-kappa B signaling pathway, and the apoptotic signaling molecule BCL2. Through the research method of network pharmacology, this study summarized the preliminary experiments of the research group and revealed the probable mechanism of FXHJ in the treatment of heart failure to a certain extent, which provided some ideas for the development of new drugs.

## 1. Introduction

Heart failure (HF) is a closed result of most cardiovascular diseases, which may be caused by damage to ventricular filling from various structural or functional disorders of the heart [1]. In observational data from Europe, the 1-year all-cause mortality rate for HF was greater than 20 percent [2]. In search of a cheap, safe, and effective drug, we turned to traditional Chinese medicine (TCM).

FXHJ is a prescription for the treatment of heart failure obtained by Dr. Xue through the combination of clinical experience and literature data. It is composed of Aconiti Lateralis Radix Praeparata (FZ), Angelicae Sinensis Radix (DG), Phellodendri Chinrnsis Cortex (HB), Lepidii Semen Descurainiae Semen (TLZ), Epimrdii Herba (YYH), and Alisma Orientale (ZX). Preliminary clinical trials of the research group have shown that FXHJ can significantly improve cardiac function, delay left ventricular remodeling, extend the 6WMT distance, improve exercise tolerance, reduce plasma BNP level, and improve patients' quality of life [3].

Due to the multicomponent and multitarget characteristics of Traditional Chinese medicine, it is difficult for us to clearly understand its mechanism of action. Therefore, we turn our attention to network pharmacology. Network pharmacology is an emerging discipline based on the integration of systems biology, molecular biology, pharmacology, and a variety of network computing platforms in the context of the era of big data [4]. Because it is compatible with the systematic and holistic treatment concept of TCM, it breaks the shackles of traditional medicine research focusing on single ingredient, single target, and single disease, so it is widely used in the study of TCM efficacy and its mechanism of action [5]. In particular, its multipart network modularization analysis and machine learning approach to explore meridian classification provide novel insights into the relationship between traditional medicine and modern medicine [6, 7]. In addition, when exploring the relationship between drugs and diseases, it is also customary to report that drugs with known efficacy can reverse predict the pathogenesis of diseases [8]—at the same time, combining computer network analysis algorithm to simulate and predict the mechanism of action of drugs. It can better display the advantages and characteristics of the systematicness of traditional Chinese medicine [9].

The mechanism of multicomponent and multitarget action of TCM has hindered the establishment of an evidence-based model of TCM and the study of toxic and side effects. Therefore, the concept of “equivalent molecular group” is suggested in this study. The various components in FXHJ can be roughly classified into three categories in the treatment of heart failure. The first had no association with the treatment of heart failure. The second had a positive effect on the treatment of heart failure, which we called active compounds. The third had a negative effect on the treatment of heart failure. Although the three components acted differently, on the whole, their final results were affirmative. After eliminating the inactive components, we take the sum of the components of positive and negative effects and turn them into the equivalent molecular group. In short, an equivalent molecular group is a collection of disease-related components, whether they play a positive or negative role in a disease.

In this study, we used the method of network pharmacology to explore the equivalent molecular group of FXHJ through the previous experimental results and elucidated the material basis and potential mechanism of action of FXHJ in the treatment of heart failure so as to provide some ideas for the development of new drugs.

## 2. Methods

### 2.1. Data Collection and Processing

Chinese medicine systematic pharmacology database (TCMSP) (http://tcmspw.com/tcmsp.php) is an open and comprehensive database of Chinese medicine ingredients and action targets. In clinical treatment, TCM is often used by oral administration. Oral bioavailability (OB) and drug-likeness (DL), two ADME-related models, are the main variables affecting the absorption of drugs from the gastrointestinal tract. Therefore, we screened bioactive components under the conditions of OB ≥ 30% and DL ≥ 0.18. Next, we find the target from the list of compounds. At the same time, we used the PubChem database (https://pubchem.ncbi.nlm.nih.gov/) to standardize the names of active ingredients. In order to make the results concise and convenient, we utilized the Uniprot database (https://www.uniprot.org/) to to convert the protein name of the target into the gene name. Then, we take the union of all the consequences and delete the duplicates. In order to get the disease target of heart failure, we used “heart failure” as the keyword to retrieve the target of heart failure in the Genecards database (https://www.genecards.org/). After the intersection of the target of FXHJ and the target of heart failure, we obtained the exact target of FXHJ in the treatment of heart failure.

### 2.2. Network Construction

We constructed the network diagram of “herb-compound-target” of FXHJ and the equivalent molecular group by Cytoscape 3.6.1. In the network diagram, “node” refers to the compound or target, and “edge” refers to the relationship between the compound and the target. Based on the analysis of the network diagram of the equivalent molecule group, we select the compound whose degree is greater than the average as the main component of the equivalent molecule group.

### 2.3. Enrichment Analysis

Direct targets were recorded into David 6.8 database (https://david.ncifcrf.gov/summary.jsp) to obtain KEGG signaling pathway data. We analyzed the results with *P* value less than 0.05 to obtain the results. According to the enrichment results and the preceding experimental results, we obtained the functional pathway of FXHJ in the treatment of heart failure. After that, we screened the effective targets and components in reverse according to the action pathway and obtained the equivalent molecular group.

### 2.4. Molecular Docking

AutoDock Vina_1.1.2 software was used to conduct molecular docking between major components and key targets of potential pathways so as to ensure their interaction activity. AutoDock Vina USES semiflexible molecular docking. That is, the pharmacophore is flexible while the protein remains rigid during the docking. The docking results are evaluated by semiempirical free energy function.

### 2.5. Specific Steps

1. In the PubChem website (https://pubchem.ncbi.nlm.nih.gov/), download the 3d structure of the active ingredient file (“SDF” format). Open Babel is used to hydrogenate atoms in molecular structure. Select the MMFF94 field to add the charge and minimize energy. Finally, AutoDcok Tools are utilized to convert the compound into a PDBQT format file.
2. Download the crystal structure of the key targets molecule from the PDB website (http://www.rcsb.org/).
3. AutoDock Tools were used to separate the target protein and its ligand, add hydrogen atom, calculate the electric charge, and export it to the PDBQT format file. AutoDock Tools were used to identify the size and center of the docking box. The center of the docking box was defined as the center of the protein crystal structure of the original ligand, and the size of the docking box included the key residues in the active site of the original ligand.
4. At last, Vina was utilized to connect the active ingredients with the target protein in turn, and Affinity was extracted. PyMol was utilized to analyze and plot the results.

## 3. Result

### 3.1. FXHJ Data Collection

**Figure 1:**
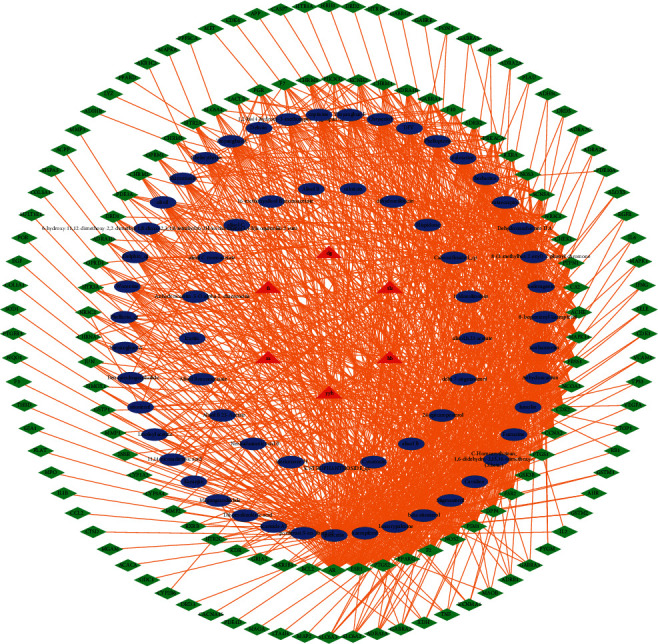
Function network diagram of FXHJ. The red triangle represents the Chinese herbs, the blue oval represents the compound, and the green diamond represents the target.

From the TCMSP database, we can get that DG has 2 blood active components and 67 predicted targets. FZ has 21 blood active components and 32 predicted targets. HB has 27 blood active components and 93 predicted targets. TLZ has 12 blood active components and 123 predicted targets. YYH has 23 blood active components and 140 predicted targets. ZX has 10 blood active components and 9 predicted targets. After adding up and deleting the duplicates of 6 Chinese herbs, we obtained 64 and 157 targets of blood components. We searched the Genecards database and obtained 10,961 targets for heart failure. Next, we took the intersection of TCM action targets and heart failure targets and obtained 144 targets. Meanwhile, we constructed the action network of FXHJ as shown in Figure 1.

### 3.2. Enrichment Analysis of Data

**Figure 2:**
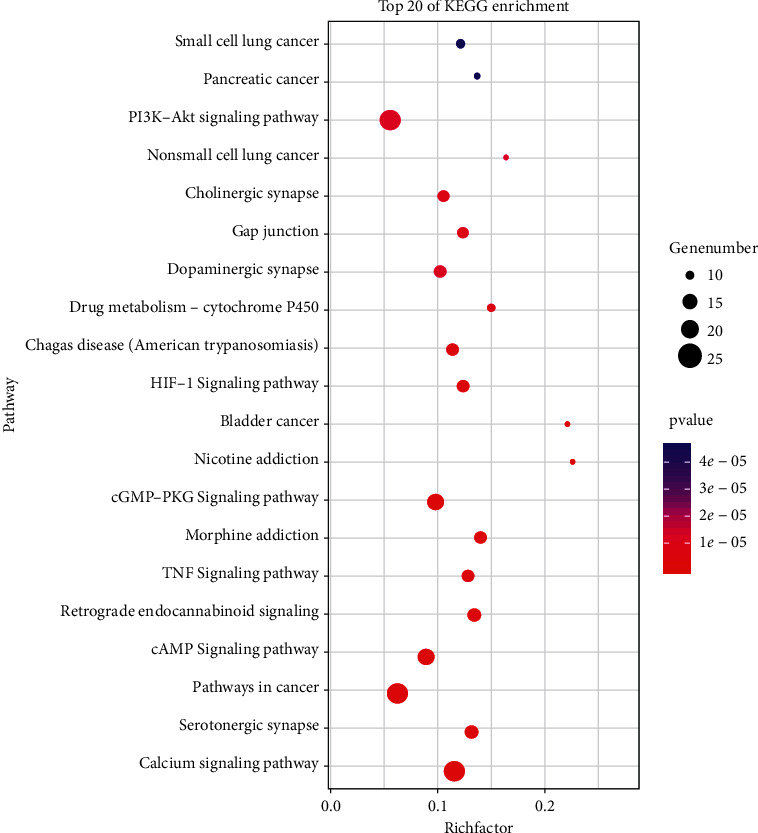
The top 20 of enrichment results. Among them, pi3k-akt signaling pathway, TNF signaling pathway, cAMP signaling pathway, and the BCL2 targets in multiple cancer pathways have been confirmed by previous experiments.

The enrichment analysis showed that the key target of FXHJ in the treatment of heart failure was concentrated in 113 pathways, among which 104 were with *P* value less than 0.05. The top 20 bits of the enrichment results are shown in Figure 2. In addition to the pathways and targets previously demonstrated by the team, the results showed that the toll-like receptor signaling pathway, VEGF signaling pathway, and NF-kappa B signaling pathway might be potential pathways for the treatment of heart failure.

### 3.3. Construction of Equivalent Molecular Clusters

**Figure 3:**
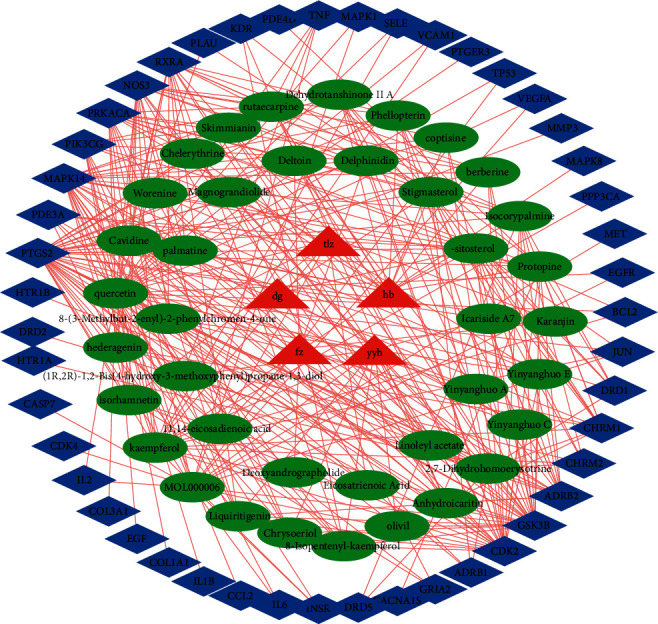
The action network diagram of the equivalent molecular group. The red triangle represents the Chinese herbs, the green oval represents the compound, and the blue diamond represents the target.

According to the enrichment analysis results, 5 Chinese herbs, 39 compounds, and 47 action targets were reverse-screened. We constructed the action network diagram of the equivalent molecular group, as shown in Figure 3. From the analysis of the network diagram, we can find that the main components of the equivalent molecular group are 17 compounds. We ranked these compounds by degree value from large to small as quercetin, Kaempferol, Luteolin, pe-sitosterol, 2, 7-dihydrohomoerysotrine, Isocorypalmine, Protopine, Stigmasterol, 8-(3-methylbut-2-enyl)-2-phenylchromen-4-one, Cavidine, Anhydroicaritin, Rutaecarpine, Phellopterin, Liquiritigenin, berberine, palmatine, and isorhamnetin.

### 3.4. Molecular Docking Results

AutoDock Vina evaluates the binding ability of small molecules to proteins, mainly by affinity. Affinity less than 0 indicates that the ligand can spontaneously bind to the receptor, and the smaller the value is, the higher the binding energy is, and the easier the active component is to bind to the receptor.

We select five key targets from toll-like receptor signaling pathway, VEGF signaling pathway, and NF-kappa B signaling pathway according to the intersection of the pathway and degree value, namely VEGFA (1VPF), TNF (2AZ5), PIK3CG (2CHZ), PTGS2 (5KIR), and MAPK1 (5NHV). The docking results are shown in Table 1.

**Figure 4:**
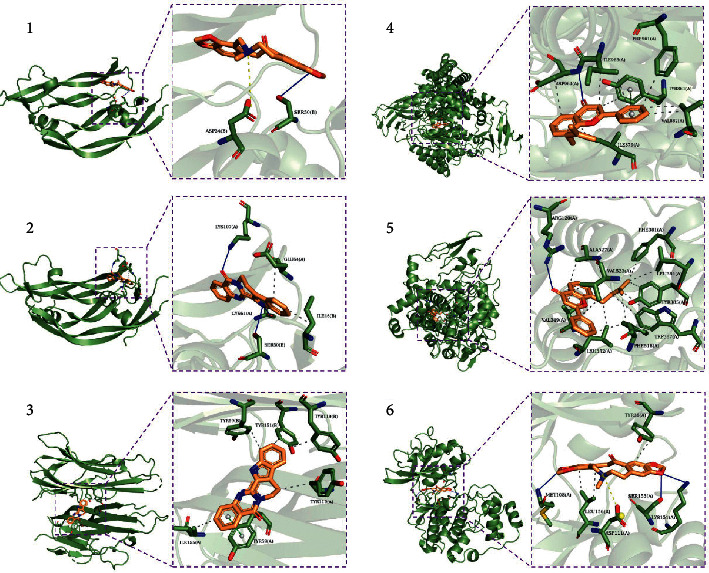
Number 1 is the combination of protopine and VEGFA. Number 2 is the combination of Rutaecarpine and VEGFA. Number 3 is the combination of Rutaecarpine and TNF. Number 4 is the combination of 8-(3-Methylbut-2-enyl)-2-phenylchromen-4-one and PIK3CG. Number 5 is the combination of 8-(3-Methylbut-2-enyl)-2-phenylchromen-4-one and PTGS2. Number 6 is the combination of protopine and MAPK1. Among them, the solid blue line represents a hydrogen bond, the dotted yellow line represents a salt bridge, the dotted gray line represents hydrophobic interaction, and the dotted green line represents pi-Stacking between benzene rings.

We visualized the combination of ligand and receptor with the highest Affinity, as shown in Figure 4. From the figure, we can see that the compound enters the target protein target active site and its binding pattern.

## 4. Discussion

In the treatment of heart failure, how to slow or even reverse ventricular remodeling has grown up to be a hot topic in recent years. Transforming growth factor beta, angiotensin II, aldosterone and endothelin, and myocardial damage or stress produce cytokines and chemokines can induce the formation of muscle fibroblasts [10, 11]. In the early stage, we have studied the relevant pathways, including the MAPK signaling pathway, the PI3KAkt signaling pathway, the cAMP signaling pathway, the TNF signaling pathway, and the apoptotic conduction molecule BCL2.

There are three subclasses in the MAPK signaling pathway, namely ERK, JNK, and p38MAPK. Cipolletta et al. demonstrated that the inhibition of p-ERK volume could delay cardiac hypertrophy and improve cardiac volume [12]. Our previous experiments showed that FXHJ could significantly reduce the ERK level of rat cardiac tissue compared with the model group (*P* < 0.01) [13].

The active form of PI3K can activate AKT, thereby promoting cell proliferation and inhibiting the apoptotic pathway. Overexpression of PI3K can cause cardiac hypertrophy. The GSK3*β* cytokine downstream of PI3KAkt, which can be inactivated by phosphorylation, is a key negative regulator of cardiac hypertrophy [14]. Our previous experiments have shown that FXHJ can reduce the expression of Pi3K and AKT, improve the expression of GSK3, and thus inhibit ventricular remodeling [15].

cAMP ACTS as excitation-coupled phosphorylation of cardiomyocyte proteins, including l-type calcium channels, sarcoplasmic reticulum ATPase 2 regulatory protein phosphor, clonidine receptor 2, phosphatase 1 inhibitors, and various contractile proteins [16]. Lin B et al. demonstrated that activation of the cAMP signaling pathway could stabilize cardiac function in rats with heart failure [17]. We studied the *β*1-AR-cAMP-PKA pathway in previous experiments and found that compared with the model group, both the FXHJ group and the captopril group could reduce the plasma cAMP level, increase the cAMP reserve of cardiomyocytes, improve cardiac function, and delay ventricular remodeling [18].

Tumor necrosis factor *α* (TNF-*α*) and the apoptotic signaling molecule BCL2 play important roles in the progression of heart failure. Liu W et al.'s serum examination of patients with chronic heart failure found that, compared with the healthy control group, the serum TNF in patients with heart failure was significantly increased, while bcl-2 was significantly decreased [19]. Studies have shown that increased TNF-*α* levels exacerbate heart failure. The optimal use of diuretics, ACE inhibitors, beta-blockers, and standard treatment with digoxin for heart failure can significantly reduce circulating TNF levels [20]. Siltanen A's experiments showed that BCL2 gene transplantation in rats with chronic heart failure could effectively improve cardiac function, enhance paracrine angiogenic signals, and promote cell proliferation and survival in failing myocardium [21]. Our previous animal experiments showed that FXHJ could effectively reduce the level of TNF-*α* in rats with heart failure, improve the expression of the BCL2 gene, and thus inhibit a variety of myocardial cell apoptosis pathways [22, 23].

In addition, through the research method of network pharmacology, we analyzed and found that the mechanism of action of FXHJ in the treatment of heart failure may also be related to the toll-like receptor signaling pathway, VEGF signaling pathway, and NF-kappa B signaling pathway. Studies have shown that 8 weeks after knocking out NF-kappa B subunit P50/NF–B1 in mice with myocardial infarction, the degree of ventricular expansion is mild, and ventricular systolic function is preserved. Therefore, the absence of the NF-kappa B subunit P50 can improve the prognosis of heart failure caused by myocardial infarction. At the same time, inhibition of the NF-kappa B signaling pathway can inhibit ventricular remodeling to a certain extent, which may be related to the reduction of preinflammatory response and the regulation of extracellular matrix [24]. Inhibiting the activation of NF-kappa B65 can prevent myocardial fibrosis and protect cardiac function by reducing the inflammatory response [25]. Vascular endothelial growth factor (VEGF) plays an important role in mediating normal cardiac function by maintaining vascular homeostasis. VEGF maintains vascular homeostasis mainly through the following mechanisms: Improve the sensitivity of blood vessels to nerve response, improve the permeability of blood vessels, promote the generation and stability of new blood vessels, and recruit stem cells and promote their homing [26]. The research of Meng-Ying He showed that when the VEGF signaling pathway was inhibited, it would lead to the imbalance of vascular homeostasis, which would lead to the generation of heart failure [27]. Toll-like receptor signaling pathway mediates the occurrence of various myocardial injuries, such as myocardial remodeling and myocardial ischemia reperfusion injury [28]. Animal studies have shown that the symptoms of myocardial ischemia in mice with toll-like receptor knockout are reduced. At the same time, toll-like receptor inhibitors could reduce myocardial ischemia in myocardial ischemia model mice [29, 30].

The analysis of the equivalent molecular group shows that there is no ZX in it. This is because the active components of ZX are mainly Alisol A 24-acetate and Alisol B, which have similar structures to aldosterone and its antagonists. They compete for aldosterone receptors to inhibit reabsorption in different parts of the tubules, inhibiting water reabsorption and increasing urine output [31]. This belongs to the category of diuretics, which is different from the screening scope of this study.

Some of the components in the equivalent molecular group have been studied experimentally, which can provide some reference for the further selection of the components. Moon DO et al. demonstrated that *β*-sitosterol promotes apoptosis by regulating the ERK and PI3KAkt signaling pathways [32], which is not conducive to the treatment of heart failure. Animal experiments on Kaempferol have shown that Kaempferol can treat heart failure by inhibiting the MAPK signaling pathway, NF-kappa B signaling pathway, and PI3KAkt signaling pathway, activating the VEGF signaling pathway and upregulating the expression of GSK3*β* [33–37]. Min Z's experiment proved that quercetin could significantly reduce the phosphorylation of ERK and inhibit myocardial cell fibrosis [38]. Meanwhile, quercetin can also treat heart failure by upregulating the expression of GSK3*β* factor and cAMP and inhibiting the expression of the NF-kappa B signaling pathway [39–41]. Although animal studies and clinical trials have shown that Luteolin can improve myocardial fibrosis and protect cardiac function by reducing myocardial oxidative stress [42, 43], other studies have shown that Luteolin can affect angiogenesis by inhibiting the VEGF signaling pathway [44, 45]. This suggests that more trials are needed to confirm whether Luteolin has side effects in patients with heart failure. Rutaecarpine has been shown to protect cardiomyocytes by inhibiting the NADPH oxidase-ROS pathway [46], a mechanism not identified in our study. Similarly, animal studies have shown that berberine can protect cardiac myocytes by upregulating the expression of BCL2 and activating AMPK and eNOS signaling pathways [47, 48]. Gao L's experiment showed that Isorhamnetin could inhibit cardiac hypertrophy by blocking the pi3k-akt signaling pathway [49].

Results of molecular docking showed that the Affinity of ligand and receptor was higher. This indicated that the ligand could well bind to the receptor, thus inhibiting the expression of this pathway. As mentioned above, inhibiting these three pathways can delay the progression of heart failure.

Interestingly, it can be evident from Figure 2 that the enrichment of tumor-related pathways is relatively high. Most of the genes enriched in these pathways are related to cell proliferation, survival, angiogenesis, and inflammatory factors, mainly involving genes such as PIK3CG, MAPK1, BCL2, NOS3, EGF, etc. These genes have been linked to cancer [50–52]. Studies have shown that tumor growth is associated with increased levels of inflammatory cytokines and a significant decrease in ventricular systolic function [53]. We know that heart failure has the same risk factors as cancer. Patients with heart failure have a higher rate of cancer than healthy control populations [54]. We also know that modern anticancer therapies are often associated with cardiotoxicity, which exacerbates or accelerates acute or chronic heart failure [55]. It may be that a combination of traditional Chinese medicine can reduce the risk of cancer in patients with heart failure [56], but more experiments and studies are needed to confirm this view.

However, there were a few shortcomings in this study. Due to limited funds, this study did not obtain the blood composition of FXHJ by mass spectrometry but selected database screening, which may cause certain errors. Secondly, although animal experiments and clinical trials have shown that FXHJ has a beneficial therapeutic effect, there is still a lack of experimental research on some components. Further research is needed to determine whether these components will cause side effects.

In summary, this study revealed the mechanism of FXHJ in the treatment of heart failure through the research method of network pharmacology and summarized the previous experimental results of the research group. It wishes to point out the direction for new drug development and further research. At the same time, the concept of the equivalent molecular group provides innovative ideas for the transformation and research of the modernization of TCM.

## Figures and Tables

**Table 1:** The figures in the table represent the affinity of molecular docking (kcal/mol). The red figure indicates that the compound has the highest affinity to the target.

Compound	VEGFA	TNF	PIK3CG	PTGS2	MAPK1
Protopine	−7.8	−9.2	−9.4	−5.4	−9.5
Cavidine	−7.4	−8.4	−8.6	−5.1	−9.3
Rutaecarpine	−7.8	−9.8	−9.2	−8.9	−9.1
8-(3-Methylbut-2-enyl)-2-phenylchromen-4-one	−7.5	−8.7	−9.6	−10.7	−9
Luteolin	−7.2	−7.8	−8.8	−9.7	−8.6
Quercetin	−7.2	−7.2	−7.7	−9.7	−8.6
Isorhamnetin	−7.2	−7.3	−7.8	−9.7	−8.5
Berberine	−7.2	−8.6	−8.5	−6	−8.5
Liquiritigenin	−7.3	−7.6	−8.8	−9.3	−8.4
Kaempferol	−6.9	−6.9	−7.5	−9.8	−8.3
Stigmasterol	−6.9	−8.3	−8.3	−7.9	−8.2
Phellopterin	−6.5	−7.4	−8	−8.3	−8.1
Palmatine	−6.2	−8	−7.5	−5.2	−7.8
Isocorypalmine	−7	−7.8	−7.7	−4.9	−7.7
Anhydroicaritin	−7.7	−8	−8.6	6.6	−7.5
2-7-Dihydrohomoerysotrine	−6	−7.4	−7.1	−2.8	−6.3

## Data Availability

The data used to support the findings of this study are available from the corresponding author upon request.
